# Effect of Monospecific and Mixed Sea-Buckthorn (*Hippophae rhamnoides*) Plantations on the Structure and Activity of Soil Microbial Communities

**DOI:** 10.1371/journal.pone.0117505

**Published:** 2015-02-06

**Authors:** Xuan Yu, Xu Liu, Zhong Zhao, Jinliang Liu, Shunxiang Zhang

**Affiliations:** 1 Department of Forestry, College of Forestry, Northwest A&F University, Yangling, China; 2 Key Laboratory of Environment and Ecology in Western China, Ministry of Education, Yangling, China; 3 College of Enology, Northwest A&F University, Yangling, China; Tennessee State University, UNITED STATES

## Abstract

This study aims to evaluate the effect of different afforestation models on soil microbial composition in the Loess Plateau in China. In particular, we determined soil physicochemical properties, enzyme activities, and microbial community structures in the top 0 cm to 10 cm soil underneath a pure *Hippophae rhamnoides* (SS) stand and three mixed stands, namely, *H. rhamnoides* and *Robinia pseucdoacacia* (SC), *H. rhamnoides* and *Pinus tabulaeformis* (SY), and *H. rhamnoides* and *Platycladus orientalis* (SB). Results showed that total organic carbon (TOC), total nitrogen, and ammonium (NH_4_^+^) contents were higher in SY and SB than in SS. The total microbial biomass, bacterial biomass, and Gram^+^ biomass of the three mixed stands were significantly higher than those of the pure stand. However, no significant difference was found in fungal biomass. Correlation analysis suggested that soil microbial communities are significantly and positively correlated with some chemical parameters of soil, such as TOC, total phosphorus, total potassium, available phosphorus, NH_4_^+^ content, nitrate content (NH_3_^−^), and the enzyme activities of urease, peroxidase, and phosphatase. Principal component analysis showed that the microbial community structures of SB and SS could clearly be discriminated from each other and from the others, whereas SY and SC were similar. In conclusion, tree species indirectly but significantly affect soil microbial communities and enzyme activities through soil physicochemical properties. In addition, mixing *P. tabulaeformis* or *P. orientalis* in *H. rhamnoides* plantations is a suitable afforestation model in the Loess Plateau, because of significant positive effects on soil nutrient conditions, microbial community, and enzyme activities over pure plantations.

## Introduction

A forest ecosystem is an essential terrestrial ecosystems that provides valuable natural resources [1]; moreover, it is involved in maintaining the quality and sustainability of our environment [2,3]. Soil has a central ecological role in forest ecosystems; it is home to many organisms and exhibits various chemical processes, which can be influenced by the diversity of plants growing aboveground [4,5]. Previous studies have shown that plant community composition can change soil pH, total organic carbon (TOC), total phosphorus (TP), total nitrogen (TN), and phenolic concentrations [6–8].

Soil microbial communities have an important role in numerous soil functions, including organic matter decomposition and nutrient cycling [9–11]. Generally, extracellular enzymes are secreted by soil microorganism in order to decompose large, polymeric compounds [12,13] and are related closely to the cycling of carbon, nitrogen, and phosphorus [14]. In addition, polyphenol oxidase has been recently reported as a key enzyme in carbon cycling [15]. Based on these findings, soil enzyme activities can function as sensitive indicators of soil microbial functionality, which potentially predicts disturbance and stress in soil microbial communities. Thus, research on the effect of tree species on soil microbial communities or enzyme activities is increasing. Thus far, many studies have demonstrated that plant diversity can alter microbial composition [16–19]. However, Fang et al. [20] investigated enzyme activities and microbial biomass in the rhizosphere under different tree compositions and found that a highly productive or keystone plant species in a community exert greater influence on soil functions than plant diversity. Furthermore, Thoms [21] concluded that abiotic factors strongly influence microbial communities. These studies suggested that the relationships among plant composition, soil microbial community, and functionality are still not understood well. The effect of aboveground plants on soil microbial composition must be further analyzed.

The Loess Plateau in China, which lies in the upper and middle courses of the Yellow River, is known to experience serious soil erosion and drought [22]. In recent years, the eco-environment of the Loess Plateau suffered from severe destruction and deterioration resulting from grassland degradation, soil erosion, and desertification because of long-term improper land-use practices. The Chinese government implemented a project called “Grain for Green” to mitigate or even eliminate the aforementioned predicament by converting large areas of farmland into forestland in the loess hilly region [23]. Sea-buckthorn (*Hippophae rhamnoides*) is widely planted as pure shrub lands in the loess hilly region because this indigenous tree species can provide rich root nodules and effective nitrogen fixation. Intensive research has demonstrated that pure *H*. *rhamnoides* shrublands have important effects on mitigating soil erosion and improving soil conditions. However, the growth and development of this species are unsatisfactory [24–26]. Thus, interest in mixed *H*. *rhamnoides* plantations has been increasing [27]. However, the link between tree species and soil microbial composition in pure or mixed forests has not yet been illustrated. The aim of the present study is to investigate the effect of four plantation models on soil microbial communities and to further analyze the relationships among tree species, soil properties, and soil microbial community structures.

## Materials and Methods

### Study sites

The experiment was conducted at the Huai Ping forest region, Shaanxi Province, China. The area is located on the central–western Loess Plateau (34°29ʹ to 34°59ʹ N, 107°56ʹ to 108°20ʹE) at 1116 m to 1276 m above sea level. Climate is classified as temperate continental monsoon, with an annual average temperature of 10.8°C. The annual rainfall is 601.6 mm and the frost-free period is 210 d. Soil is classified as cinnamon soil according to the national standards of China: China soil classification and code (GB/T 17296–2009).

The experiment site lies on a relatively flat terrain in the loess hilly gully region. We chose four plots, which represent four tree diversities. Among these plots, three were mixed plantations, namely, *H*. *rhamnoides* and *Robinia pseucdoacacia* (SC), *H*. *rhamnoides* and *Pinus tabulaeformis* (SY), and *H*. *rhamnoides* and *Platycladus orientalis* (SB). The mixed proportion of *H*. *rhamnoides* and other tree species was 1:1. The fourth plot was pure *H*. *rhamnoides* plantation. All plots were established following a spacing of 1 m × 1 m. These plots are adjacent and are 150 m apart from one another. The site conditions, including climatic characteristics, soil properties, textures, and mineral compositions were similar in the four plots [28].

### Soil sampling

In July 2013, we randomly selected three subplots (20 m × 20 m) as the three replicates in each plot. The soil samples were obtained from the topsoil (0 cm to 10 cm) by using a soil corer (diameter: 5 cm), after litter was excluded. In each subplot, five soil cores were collected and mixed into one sample. A total of nine soil samples were collected for further analysis. The soil samples were placed in plastic bags and immediately transported to the laboratory in an icebox. All soil samples were sieved (2 mm). A portion of each soil sample was sieved and then temporarily stored at 4°C less than 2 hours for microbial community structure analysis. The lipid extraction was immediately carried out after all the samples were sieved. Another portion was air-dried for soil chemical property and enzyme activity analyses.

### Chemical properties of soil

Soil pH, TOC, TN, carbon nitrogen ratio (C/N), TP, total potassium (TK), available phosphorus (AP), available potassium (AK), NH_4_^+^ content, and NO_3_^−^ content were measured according to the method described by Liu [29].

### Phospholipid fatty acid analysis (PLFA)

The composition of soil microbial community was evaluated by measuring fatty acid methyl ester (FAME). The procedures for lipid extraction and PLFA were based on Frostegard et al. [30] and White et al. [31], respectively. Lipids were extracted from 8 g soil from each sample in a monophasic solution of chloroform, methanol, and citrate buffer (1.0:2.0:0.8 v/v/v) [32]. Phospholipids were separated from glycolipids and netural lipids on a silicic acid column, and subsequently converted into fatty acid methyl esters through mild alkaline methanolysis.

Finally, fatty acid methyl esters were analyzed on an Agilent 6850N gas chromatographer (Agilent Technologies, Palo Alto, USA) with an ULTRA-2 column (length: 25 m, internal diameter: 0.20 mm, and film thickness: 0.33 μm). Methyl nonadecanoate (Me19:0) was used as the internal standard for calculating FAME concentrations. Fatty acid peaks were identified according to retention time and mass spectrum information. Concentrations of individual PLFA (nmol lipid g^−1^ soil) were calculated by comparing peak areas with the internal standard [33].

In total, the sum of PLFAs, i.e., 14:0, 15:0, 17:0, i14:0, a15:0, i15:0, i16:0, a17:0, cy17:0, and 18:1w5c, was used to evaluate total bacteria biomass. Among these, fatty acids i14:0, a15:0, i15:0, i16:0, and a17:0 were used as indicators for Gram-positive bacteria (G^+^) [34]; cy17:0 and 18:1w5c for Gram-negative (G^−^) bacteria [35,36]; and 14:0, 15:0, and 17:0 for general bacteria [37]. PLFAs 10Me17:0 and 10Me18:0 were used as indicators for actinobacteria [38]. Fungi markers included 18:1w9c, 18:3w6c, and 20:1w9c [39,40]. The ratio of G^+^ biomass and G^−^ biomass were calculated.

### Soil enzyme activities

The potential activities of five extracellular enzymes were used to assess the microbial community function. The test methods used were those described by Guan [41]. Alkaline phosphatase, invertase, and urease were determined in 5 g soil. The activities of polyphenol oxidase and peroxidase were determined in 1 g soil. The substrates were disodium phenylphosphate, sucrose, urea, and pyrogallol. The controls used distilled water instead of substrates. All five enzyme activities were determined via the colorimetric method and expressed based on soil dry matter. Each enzyme had three replicates.

### Statistical analysis

We evaluated the significant differences of the selected parameters using one-way ANOVA followed by least significant difference (LSD) tests (*p* < 0.05). The correlations between soil microbial community structure and certain measurable factors were obtained via Pearson’s *r* with *p* < 0.05 significance threshold (two-tailed test). Principal component analysis (PCA) was performed to explain the relationship between soil microbial community structures based on the amounts of individual PLFA and tree species. All data were analyzed with the SPSS package (version 11.0).

## Results

### Soil chemical characteristics

As shown in Table 1, soil pH, TP, AP, and NO_3_^−^ contents of the three mixed plantations were significantly higher than those of the pure plantation. TOC, TN, NH_4_^+^, and AK contents significantly increased in SY and SB, but not in SS. However, mixed plantation SC exhibited no significant advantage in the aforementioned parameters over the pure plantation.

**Table 1:** Soil chemical characteristics in different plantations.

Site	pH	TOC (g·kg^−1^)	C/N ratio	TN (g·kg^−1^)	TP (g·kg^−1^)	TK (g·kg^−1^)	NH_4_^+^ (g·kg^−1^)	NO_3_^-^ (g·kg^−1^)	AP (g·kg^−1^)	AK (g·kg^−1^)
SS^a^	7.72d^b^ (0.09)^c^	11.93b (1.72)	10.55b (0.96)	1.13b (0.10)	0.38b (0.04)	12.74b (0.86)	4.77b (0.39)	1.03c (0.13)	9.27b (0.19)	102.03b (5.00)
SY	8.14b (0.04)	24.70a (2.88)	16.32a (3.80)	1.54a (0.19)	0.72a (0.14)	16.76a (0.35)	8.66a (0.45)	1.42b (0.16)	15.27a (0.15)	139.12a (3.73)
SB	8.27a (0.07)	22.07a (1.66)	14.88ab (2.65)	1.50a (0.16)	0.73a (0.10)	16.34a (1.32)	7.41a (0.61)	1.92a (0.06)	15.60a (0.14)	126.35a (4.27)
SC	7.99c (0.04)	15.70b (1.55)	14.13ab (3.21)	1.14b (0.20)	0.71a (0.07)	16.21a (0.80)	4.91b (0.85)	1.33b (0.09)	14.44a (0.06)	108.71b (6.14)

^a^ SS: pure *Hippophae rhamnoides* plantation; SY: mixed plantation with *Hippophae rhamnoides* and *Pinus tabulaeformis*; SB: mixed plantation with *Hippophae rhamnoides* and *Platycladus orientalis*; SC: mixed plantation with *Hippophae rhamnoides* and *Robinia pseucdoacacia*.

^b^ Different letters in the columns indicate significant differences at *p* < 0.05 levels via the LSD test.

^c^ Standard deviation.

### Microbial community structure by using PLFAs

The microbial community structure of the four models is presented in Table 2. Bacteria biomass and Gram^+^ bacteria biomass were significantly higher (*p* < 0.05) in SB, SY, and SC than in SS. Meanwhile, Gram^−^ bacteria biomass was higher in SY and SB than in SS. However, no significant difference was found between SC and SS. In addition, no significant difference was observed in the four plantation models with regard to fungi biomass. Gram^+^ to Gram^−^ ratio was lowest in SS. Nevertheless, no significant difference was determined among SB, SY, and SC.

**Table 2:** Soil microbial community structure based on indicator lipids (n·mol g^−1^ soil) from the four sites.

Site	Total lipid^**b**^	Bacteria	G^+^ ^**d**^	G^−^	Fungi	Ratio G^+^/G^–^
SS^a^	12.15c^c^ (0.10)^e^	3.93c (0.64)	2.99b (0.44)	0.61c (0.15)	1.56a (0.02)	5.00a (0.44)
SY	19.57ab (1.25)	7.63ab (1.35)	5.97a (1.09)	1.08ab (0.13)	1.56a (0.39)	5.51a (0.44)
SB	23.90a (1.56)	9.15a (1.32)	6.50a (0.90)	1.42a (0.33)	2.11a (0.39)	4.91a (0.74)
SC	17.93b (2.28)	6.78b (1.15)	5.26a (0.93)	0.96bc (0.13)	1.53a (0.17)	5.45a (0.20)

Soil microbial community structure based on indicator lipids (nmol g^−1^ soil) from the four sites

^a^ SS: pure *Hippophae rhamnoides* plantation; SY: mixed plantation with *Hippophae rhamnoides* and *Pinus tabulaeformis*; SB: mixed plantation with *Hippophae rhamnoides* and *Platycladus orientalis*; SC: mixed plantation with *Hippophae rhamnoides* and *Robinia pseucdoacacia*.

^b^ Total lipid was the sum of the 31 detected fatty acids.

^c^ The values were the means of three replicates. Different letters within columns indicate significant differences at *p* < 0.05 levels through the LSD test.

^d^ G^+^ and G^−^ represent gram-positive and gram-negative bacteria, respectively. G^+^/G^−^ was the ratio of the sum of gram-positive bacteria to the sum of gram-negative bacteria.

^e^ Standard deviation.

**Fig 1:**
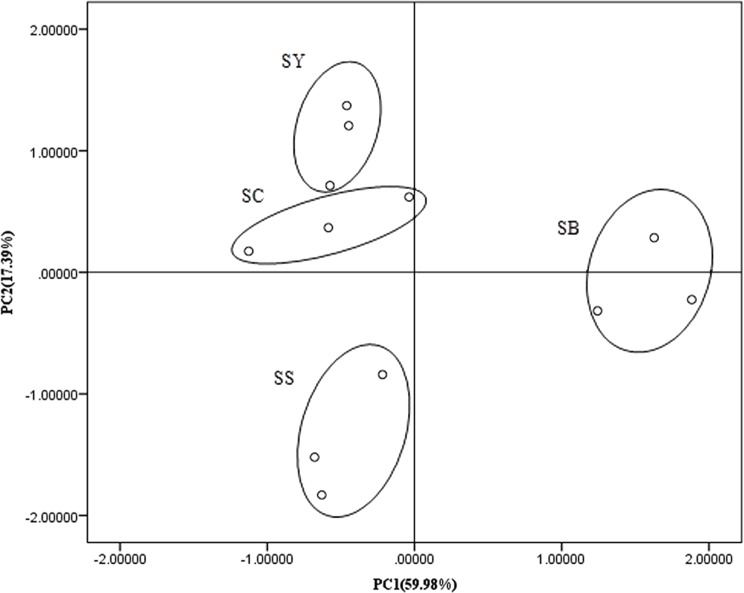
PCA of fatty acids from different plantations models. SS: pure *Hippophae rhamnoides* plantation; SY: mixed plantation with *Hippophae rhamnoides* and *Pinus tabulaeformis*; SB: mixed plantation with *Hippophae rhamnoides* and *Platycladus orientalis*; SC: mixed plantation with *Hippophae rhamnoides* and *Robinia pseucdoacacia*.

PCA with PLFA data was used to assess the differences in microbial community structure among all plots. As shown in Fig. 1, PCA can discriminate microbial composition among different plantations. Principal component 1 (PC1) can explain 59.98% of the total variance. Fatty acids i13:0, 18:3w6c, 20:1w9c, 16:1w5c, 18:1w9c, i14:0, and 17:1w8c exhibited higher loadings in PC1. Principal component 2 (PC2) can explain 17.39% of the total variance. Fatty acids 18:0 10Me; c19:0, i16:0, 16:1 2OH, a17:0, 17:0 10Me, a15:0, i16:1, and 18:1 2OH exhibited higher loadings in PC2. These results suggest that the microbial communities of SB and SS models were distinguished from each other and from SY and SC through PC1 and PC2. Moreover, the microbial community structure of SS was different from those of the three mixed stands. These results demonstrate that tree species may have an important role in soil microbial composition.

### Soil enzyme activities

Significant differences in soil enzyme activities were determined from different tree species (Table 3). In terms of invertase and urease activities, the three mixed *H*. *rhamnoides* plantations presented higher values than the pure *H*. *rhamnoides* plantation (SS) (*p* < 0.05). With regard to phosphatase and peroxidase activities, no significant difference was found between SS and SC. In contrast to the other four enzyme activities, polyphenol oxidase activity in SS was higher.

**Table 3:** Soil enzyme activities from different plantations.

Site	Invertase (mg·g^−1^·d^−1^)	Urease (mg·g^−1^·d^−1^)	Phosphatase (mg·g^−1^·d^−1^)	Polyphenol oxidase (mg·g^−1^)	Peroxidase (mg·g^−1^)
SS^a^	2.82c^b^ (0.15)^c^	0.31c (0.18)	0.53b (0.06)	1.73a (0.11)	0.82b (0.15)
SY	3.77a (0.37)	0.56a (0.12)	0.92a (0.25)	1.24b (0.11)	1.55a (0.20)
SB	3.52ab (0.25)	0.52a (0.06)	0.85a (0.19)	1.03b (0.26)	1.44a (0.31)
SC	3.13bc (0.44)	0.42b (0.04)	0.54b (0.30)	1.65a (0.20)	1.08b (0.05)

^a^ SS: pure *Hippophae rhamnoides* plantation; SY: mixed plantation with *Hippophae rhamnoides* and *Pinus tabulaeformis*; SB: mixed plantation with *Hippophae rhamnoides* and *Platycladus orientalis*; SC: mixed plantation with *Hippophae rhamnoides* and *Robinia pseucdoacacia*.

^b^ Different letters within columns indicate significant differences at *p* < 0.05 levels through the LSD test.

^c^ Standard deviation.

### Correlation analysis among microbial communities, soil properties, and enzyme activities

Total PLFAs, Gram-positive bacteria biomass, and Gram-negative bacteria biomass exhibited significant positive correlations with all tested soil characteristics (Table 4). Bacteria biomass only positively correlated with TOC, TP, TK, AP, and NO_3_^−^content (*p* < 0.05). However, no significant relationship was found between fungal biomass and all soil properties. In addition, the correlation coefficients between the biomass of the PLFA groups and soil enzyme activities are shown in Table 5. Total PLFAs, bacteria biomass, gram-positive bacteria biomass, and gram-negative bacteria biomass positively correlated with urease and peroxidase (*p* < 0.05), but negatively correlated with polyphenol oxidase activities. Fungal biomass showed no significant correlation with all tested enzyme activities.

**Table 4:** Correlation coefficients between the biomass of the PLFA groups and soil properties.

	TOC^**a**^	TN	TP	TK	AP	AK	NH4^+^	NO_3_^−^	C/N
Total lipids	0.822**^b^	0.814*	0.895**	0.913*	0.956**	0.824**	0.787**	0.888**	0.802**
Bacteria	0.868**	0.403	0.608*	0.664*	0.738*	0.491	0.416	0.746**	0.283
G^+^	0.765**	0.640*	0.726**	0.859**	0.859**	0.662*	0.626*	0.795**	0.596*
G^−^	0.722**	0.672*	0.724**	0.796**	0.816**	0.627*	0.629*	0.846*	0.582*
Fungi	0.129	0.212	0.039	0.264	0.171	0.132	0.202	0.209	0.073

^a^ TOC: total organic carbon; C/N: carbon nitrogen ratio; TN: total nitrogen; TP: total phosphorus; TK: total potassium; NH_4_^+^: ammonium content; NO_3_^−^: nitrate content; AP: available phosphorus; AK: available potassium.

^b *^ and **denote significant differences at *p* < 0.05 and *p* < 0.01, respectively.

**Table 5:** Correlation coefficients between the biomass of the PLFA groups and soil enzyme activities.

	Urease	Invertase	Phosphatase	Polyphenol oxidase	Peroxidase
Total lipids	0.898**^a^	0.795*	0.630*	−0.820**	0.796**
Bacteria	0.811**	0.550	0.571	−0.681*	0.706*
G^+^	0.816**	0.656*	0.666*	−0.771*	0.646*
G^−^	0.837*	0.518	0.472	−0.741*	0.669*
Fungi	0.238	−0.035	0.198	−0.277	0.057

a * and **denote significant differences at *p* < 0.05 and *p* < 0.01, respectively.

## Discussion

Soil organic carbon is a major component of the soil carbon pool, which depends on the balance between carbon input through litter fall and rhizodeposition as well as the release of carbon during decomposition [42]. Previous studies have revealed that forest management practices, such as fertilization [43], tillage [44], and reconstruction [45] can affect soil carbon dynamics and storage. In the present experiment, the results showed that soil organic carbon, NH_4_^+^ content, and NO_3_^-^ content were significantly higher in mixed plantations than in pure *H*. *rhamnoides* plantation. The higher soil organic carbon, NH_4_^+^ and NO_3_^-^ content were also detected in mixed *Cunninghamia lanceolata* and *Michelia macclurei* [46], *Pinus nigra* and *Quercus ilex* [47] plantation. *H*. *rhamnoides* is a nitrogen-fixing species [48]. According to Vitousek [49], nitrogen-fixing trees can increase soil organic carbon by enhancing soil nitrogen availability and improving soil conditions. The present experiment illustrated that the interaction of *H*. *rhamnoides* and *P*. *tabulaeformis* or *P*. *orientalis* can have a synergistic effect on improving soil nitrogen availability. It is assumed that these plants could absorb nitrogen nutrition which promotes plant growth and increase plant productivity, amount, and plant litter quality [50]. The decomposition of plant litter is an important source of soil organic input. Therefore, the mixed plantations of *H*. *rhamnoides* and *P*. *tabulaeformis* or *P*. *orientalis* may increase soil carbon sequestration, and thus, affect soil carbon storage.

Soil microorganisms excrete soil enzymes to drive mineralization and decomposition [13, 51–52]. These microorganisms are directly responsible for the initial processing of nutrient cycling and the variation of vegetation communities [53]. Therefore, soil enzyme activities can be an evaluation strategy for microbial mineralization processes. Extracellular enzyme activities may be related to the element (i.e., carbon, nitrogen, and phosphorus) cycling in soil. In the present study, we tested soil enzyme activities (phosphatase, urease, invertase, phenol oxidase, and preoxidase) with respect to the cycling of carbon, nitrogen, and phosphorus to evaluate microbial mineralization processes. The results showed that the activities of phosphatase and urease were significantly higher in SY and SB than in SS. This result suggests that the introduction of *P*. *tabulaeformis* or *P*. *orientalis* may accelerate the mineralization rates of soil organic phosphorus and nitrogen. In addition, phenol oxidase was effective in degrading complex materials (e.g., lignin) and typically correlated with high decomposition and mass loss rates in soil organic matter [15]. In the present experiment, we also found that the activities of phenol oxidase were significantly high in SS. However, soil organic matter was low.

The influence of soil microbial communities on land-use changes or disturbances can be estimated with community-level PLFA profiles [54–56]. In the present study, total PLFAs were significantly higher when *H*. *rhamnoides* was mixed with *P*. *tabulaeformis* or *P*. *orientalis*. Furthermore, microbial communities of the three mixed plantations were clearly separated from the pure stand by PCA. Tree species have an important role in soil microbial community structure. These findings are consistent with many previous studies that determined the effects of tree species on soil microbial community composition [46,57–59]. However, some reports showed that tree species have an insignificant effect on microbial community composition [17,60]. These reports may suggest that apart from plant species, specific environmental factors have significant effects on soil microbial community structure. These factors can affect plant growth, and thus, enhance or weaken the effects of plant species on soil microbial community structure.

Vegetation composition can affect soil organic matter accumulation, acidity, and substrate quality (e.g., TOC, TN, TP, and C/N ratio) by regulating the quality and quantity of litter and plant root exudate composition [61,62]. The present study showed that some soil properties were significantly higher in mixed *H*. *rhamnoides* plantations (SY and SB) than in the pure plantation (SS). This result can reaffirm that soil properties are influenced by tree species diversity. Correlation analysis revealed that soil properties are positively correlated with total lipid and bacteria abundance. These findings suggest that soil properties may have an important role in determining soil microbial community, which agrees with some previous studies [63,64]. In addition, forest litter and roots are the main sources of soil organic matter. Furthermore, the fungi (i.e. ectomycorrhizal fungi) [65] and other organisms (i.e. protozoa) [66] could also contribute to soil organic matter. Soil organic matter can provide nutrition to microbial communities [67]. Therefore, plant species can indirectly affect soil microbial communities. Future studies can be conducted to measure the attributes of litter and root exudates, and evaluate their influence on the functioning of microbial communities. In conclusion, mixing plantations (SY and SB) are more suitable models which are beneficial to improve soil chemical properties, microbial community structure, and enzyme activities.

## Conclusion

Mixed plantations, i.e., *H*. *rhamnoides* and *P*. *tabulaeformis*, *H*. *rhamnoides* and *P*. *orientalis*, can have a synergistic effect on microbial community structure, enzyme activities, and soil organic carbon storage because of the higher contents of soil organic carbon, total N, available P, available K, NH_4_^+^ and NO_3_^-^ compared with monocultures. Therefore, introducing *P*. *tabulaeformis* or *P*. *orientalis* to *H*. *rhamnoides* plantations can be an alternative for increasing plant productivity and enhancing soil conditions in *H*. *rhamnoides* plantations. The highlight of the present study is finding the appropriate tree species for mixed *H*. *rhamnoides* plantations, which contributes to minimize interspecies competition and maintain soil fertility.
